# Biological effects of radiation on cancer cells

**DOI:** 10.1186/s40779-018-0167-4

**Published:** 2018-06-30

**Authors:** Jin-song Wang, Hai-juan Wang, Hai-li Qian

**Affiliations:** State Key Laboratory of Molecular Oncology, National Cancer Center/National Clinical Research Center for Cancer/Cancer Hospital, Chinese Academy of Medical Sciences and Peking Union Medical College, RM6102, New Research Building, 17 Panjiayuan Nanli, Chaoyang District, 100021 Beijing China

**Keywords:** Radiation, Cancer cells, Biological features, Combinational therapy

## Abstract

With the development of radiotherapeutic oncology, computer technology and medical imaging technology, radiation therapy has made great progress. Research on the impact and the specific mechanism of radiation on tumors has become a central topic in cancer therapy. According to the traditional view, radiation can directly affect the structure of the DNA double helix, which in turn activates DNA damage sensors to induce apoptosis, necrosis, and aging or affects normal mitosis events and ultimately rewires various biological characteristics of neoplasm cells. In addition, irradiation damages subcellular structures, such as the cytoplasmic membrane, endoplasmic reticulum, ribosome, mitochondria, and lysosome of cancer cells to regulate various biological activities of tumor cells. Recent studies have shown that radiation can also change the tumor cell phenotype, immunogenicity and microenvironment, thereby globally altering the biological behavior of cancer cells. In this review, we focus on the effects of therapeutic radiation on the biological features of tumor cells to provide a theoretical basis for combinational therapy and inaugurate a new era in oncology.

## Background

Tumor radiotherapy is a technique that is used to inhibit and control growth, metastasis and proliferation of malignant tumor cells using various types of ionizing radiation. Over the past few decades, the development of molecular biology and experimental techniques has further elucidated the effects of radiation on the biological properties of cancer cells. During tumor treatment, radiation is considered to be a “double-edged sword” because it not only affects the proliferation, metastasis and other biological processes of neoplasms, but may also genetically modify normal tissues, causing damage to non-tumor cells, which is a detrimental effect on the body that we do not expect. Traditionally, it has been revealed that irradiation can directly affect malignant cells by affecting DNA structure stability and repair processes, triggering DNA double-strand breaks (DSBs) and inducing therapeutic effects against tumor cells, such as apoptosis, necrosis, senescence, and abnormal mitosis [1, 2].

The latest research has shown that irradiation not only disturbs the structure of neoplasm cells, such as the cell membrane and organelles but also interferes with cell signal transduction and regulation, changing neoplasm cells immunogenicity and their microenvironment [3, 4]. Additionally, irradiated cancer cells can deliver a bystander response signal to adjacent non-irradiated tumor cells, which kills adjacent neoplasm cells and protects normal tissue from damage caused by rays [5]. With regard to radiotherapy of malignant tumors, it is necessary to ensure that the correct dose is projected in the correct manner to the precise position of the patient to achieve the best possible therapeutic effect while harming normal tissue as little as possible. Since the introduction of the concept of “precision medicine” in 2011, the emphasis has been placed on individualized and accurate treatment, which are aimed at improving the effectiveness of cancer diagnosis and treatment. A better understanding of the response of malignant tumors to radiation at the molecular, cellular and tissue levels will be advantageous to form new strategies for the combined treatment of tumors.

### Radiation causes DNA damage

Apoptosis, necrosis, and senescence of cancer cells induced by DNA damage are the major effects of radiation on tumor tissue and are beneficial effects of radiation for cancer therapy. Radiation directly causes DNA damage like single-strand breaks (SSBs), DSBs, DNA crosslink and DNA-Protein crosslinks or induces damage indirectly to DNA by reactive oxygen species (ROS)/reactive nitrogen species (RNS). Of these, DSBs, an initiating factor of chromosomal rearrangements that increase in a linear-quadratic function under high dose rates (HDR) of radiation, are considered to be the most harmful lesion induced by radiation [6–9]. Quick phosphorylation of histone H2AX on serine 139 (γH2AX) is deemed to be a sensitive marker of ionizing radiation-induced DSBs [10]. Collis et al. [11] observed that decreased activation of γH2AX following low-dose-rate exposures compared with high-dose-rate radiation in cancerous and normal human cells indicating that DNA damage induced by low-dose-rate radiation might be able to be repaired efficiently. The responses of tumor cells to heavy radiation-induced DNA damage are transmitted from DNA damage sensors and cell cycle regulators and can be categorized into three stages: DNA damage induction, DNA damage signal pathway activation and the repair phase of DNA damage [2, 12].

Similar to DSBs, within a certain range, the yield and complexity of SSB and non-DSB cluster damage are positively correlated with the radiation dosage. However, DSBs are relatively unmanageable. DSBs are restored by two main pathways, homologous recombination and non-homologous end joining (NHEJ) [13, 14]. If DNA damage is renovated effectively and precisely, cells recover their normal functions; otherwise, chronic DNA damage will trigger apoptosis or cell senescence [15]. Moreover, radiation can activate protein tyrosine phosphatase non-receptor type 14 (Ptpn14) through DNA damage signaling in a mouse embryonic fibroblasts model expressing H-Ras^v12^ [16]. Activated Ptpn14 can inhibit the proliferation of pancreatic cancer cells by negative regulation of the YAP oncogene [16]. Furthermore, Jutta’s group demonstrated that HeLa cells exposed to 10Gy of X-irradiation harbored the characteristics of mitotic catastrophe and increased intra-nuclear chromosome territories compared with a control group, and thus initiating cell apoptosis [17]. However, there are still some cancer cells that can induce endopolyploidization as an escape route from cell death, which emphasizes the significance of precisely distinguishing tumor cells and their variants.

### Rays affect the performance of cancer cell organelles

#### Radiation damages the endoplasmic reticulum

Radiation-induced damage to organelles may also play an important role regarding the effects of radiation. Studies have shown that IR-induced tumor cell death is associated with endoplasmic reticulum (ER) disturbances [18]. As a major target organelle of radiation, ER is highly sensitive to changes in the internal environment. Milieu interne changes induced by radiation will cause an endoplasmic reticulum stress (ERS) response, which leaves tumor cells in a stress state. A mild ERS-activated unfolded protein response (UPR), endoplasmic reticulum overload reaction (EOR) or sterol regulatory element binding protein (SREBP) can not only initiate autophagy and remove misfolded proteins to restore ER homeostasis and promote cell survival but also stimulate the expression of protective molecules, such as endoplasmic reticulum chaperones, to protect cells from damage [19]. However, when the radiation dose is large enough, sustained and excessive ERS will have a variety of biological effects on tumor cells. The endoplasmic reticulum may cause autophagic cell death by over-activating the autophagy pathway; additionally, the ER may initiate specific apoptotic pathway to promote tumor cell apoptosis [20].

#### IR-induced ribosomal changes

Ribosomes, a type of intracellular ribonucleoprotein particle, are mainly composed of RNA (rRNA) and proteins. Their indispensable function is to translate amino acids into polypeptide chains according to the mRNA sequence, which lays foundation for maintaining the systematical operation of bioactivities and given their multifunctional and regulatory activities, numerous studies have illustrated that ribosomes play important roles in the initiation and development of cancer [21–23]. HyeSook’s group indicated that 4Gy of IR dissociated the MIF-rpS3 complex (migration inhibitory factor, MIF and ribosomal protein S3, rpS3) by inducing casein kinase 2α (CK2α)-mediated rpS3 phosphorylation and that separation of MIF-rpS3 affected the NF-κB pathway, concomitantly stimulated cancer-associated inflammation and promoted metastasis of NSCLC cells [24]. Similarly, Yang et al. [25] demonstrated that CK2α- and PKC-induced phosphorylation of rpS3 and TNFR-associated factor 2 (TRAF2) granted NSCLC cells radioresistance by activating the NF-κB signaling pathway; however, CK2α- and PKC-deficient NSCLC cells are radio-responsive. Of note, an attempt to regulate rpS3 and MIF or TRAF2 in combination with radiation may have a high pharmacological therapeutic potency by retaining the normal activity of rpS3. Clinically, by analyzing the serum composition changes of 35 patients with prostate cancer before and after treatment, Ingrosso et al. [26] indicated that the ribosomal P0 protein appeared to be increased according to the degree of exposure along with a high immunogenic antigen, and consequently, its immunogenicity increased following RT, which highlights that the generation of anti-P0 autoantibodies after IR could have clinical significance.

#### Radiation affects the behavior of mitochondria

In addition to endoplasmic reticulum disorders, radiation exposure has a significant effect on the biological behavior of the mitochondria in tumor cells. Kam et al. [27] found that radiation can directly promote the release of cytochrome C (Cyt-c) by releasing ROS or by indirectly triggering Cyt-c-induced apoptosis through altering the permeability of the mitochondrial membrane both in vitro and in vivo. Cyt-c, released from the mitochondria into the cytoplasm, forms a complex with the apoptotic factor Apaf-1. Caspases-9, recruited by the CARD domain of the Cyt-c/ Apaf-1 complex, can be activated by homo-activation, which is a motivation to initiate apoptotic signaling pathways mediated by caspases-3 and caspases-7. As shown by Walsh et al. [28] via in situ live cell imaging of individual mitochondria stained with Tetramethylrhodamine ethyl ester, targeted irradiation triggers mitochondrial membrane depolarization, which can induce cytochrome c release and is involved in apoptosis. Simultaneously, Fachal et al. [29] incubated a cytoplasmic extract of irradiated cells with normal cells and found that the extract induced DNA fragmentation of the normal nucleus, illustrating that the cytoplasm might also be an important target of the radiotoxic effect. Moreover, the direct action of radiation on mitochondrial DNA (mtDNA) could activate programed cell death by itself [29]. In summary, the impact of radiation on the biological properties of tumor cell organelles also has a significant impact on the development and recurrence of cancer [30].

#### Irradiation-induced lysosomal damage

Lysosomes are important organelles of animal cells that digest various biological macromolecules in the body and involved in cellular metabolism, immunity and hormone secretion regulation and other activities. If the lysosomal membrane is damaged by environmental stress including radiation, various hydrolases in the lysosome enter the cytoplasm and disintegrate the cell. Furthermore, Lennart assessed radiation-induced lysosomal destabilization of the lymphoma J774 cell line in vitro and found that a 40Gy radiation dose triggered remarkable upregulation of intra-lysosomal labile iron, which resulted from “reparative auto-phagocytosis” secondary to radiation-induced lysosomal damage, along with an outflow of lytic enzymes as well as abnormal iron and consequent cellular injuries [31]. When different dosages of X-rays (2Gy, 4Gy and 8Gy, respectively) were given to lung adenocarcinoma A549 cells, the number of lysosomes was significantly increased, as detected by the Lyso-Tracker Red fluorescent probe, and senescence of A549 cells was further aggravated. Radiation can exacerbate the aging of cancer cells in a lysosome-related manner or not. Smooth muscle protein 22-alpha (SM22α) activates the p16INK4a/retinoblastoma signaling pathway, promoting HepG2 cell senescence, which is caused by a subclinical dose of γ-radiation (0.05 and 0.1Gy) [32]. Besides, apoptosis in UVA-irradiated keratinocytes is co-mediated by lysosomal exocytosis combined with caspase-8, indicating the essential role that lysosomes play in irradiation-induced effects. Biologically, lysosomes are indispensable sites and regulators of cancer cell autophagy processes, which are the essential inducers of tumor multidrug resistance (MDR), whereas lysosomal dysfunctionality resulting from irradiation could reverse this effect. Despite limited data on lysosome-mediated tumor therapy, the irreplaceable function of lysosome makes it a promising target for oncotherapy.

### Radiation affects the plasma membrane

The use of a micro-beam system to selectively irradiate a nuclear-free sphingo-like ceramide membrane confirmed that the plasma membrane is another target of ionizing radiation. On one side, radiation can directly affect the composition of the tumor cell membrane, such as membrane receptors, lipids and membrane proteins, which have significant effects on cell membrane permeability, integrity, and mobility. On the other side, radiation induces amounts of ROS and RNS, which affect a number of intracellular signaling pathways and regulate a variety of cell functions and structures, such as apoptosis, proliferation, cytoskeleton and morphological changes.

#### Radiation affects the cell membrane biological properties

Stability of cell membrane is instrumental to the occurrence and development of malignant neoplasms. Radiation can directly cause corrosive damage to the cell membrane or indirectly alter the biological characteristics of the cell membrane by affecting the composition of the cell membrane. When radiation acts on the cell membrane, it causes corrosive damage to the cell membrane diametrically, which affects the permeability, integrity, and mobility of the cell membrane and, eventually leads to cell disaster [33]. In addition, radiation can directly activate sphingomyelinases on the tumor cell membrane, and the destruction of polar components of tumor cells from the degradation of lipids by sphingomyelin enzymes leads to impairment of the membrane barrier, which is critical to the integrity of tumor cells [3]. Rays can also affect the intensity and activity of membrane proteins by disturbing peptide links, hydrogen bonds and disulfide linkages, which are significant for maintaining normal membrane protein structures [34, 35]. Immunologically, environment stress, including radiation, initiates translocation of heat shock protein 70 (Hsp70) from an intracellular bio-site to the extracellular milieu, where it can originate an innate immune response under the condition of pro-inflammatory cytokines or unleash adaptive immune system in the presence of tumor-derived peptides via antigen cross-presentation [36–38]. Fractionated radiation (5 × 2Gy) facilitates further Hsp70 release and membrane expression in dying cancer cells and hence augments the immune-recognization of Hsp70^+^ tumor cells by TKD/IL-2 activated NK cells [39, 40], indicating the essential roles of HSPs as targets for adaptive and innate anti-tumor immune responses. The therapeutic efficacy of united therapy mediated by intratumoral dendritic therapy and radiation rose immeasurably by a co-injection with recombinant Hsp70 demonstrated in CT26 colorectal cancer mouse models [41]. Given the importance of cell membranes to tumor cells and irradiation-induced enhanced immunotoxicity via modulating membrane elements expression, radiotherapy-combined strategies to target tumor cell membranes may be a promising treatment.

#### Radiation regulates cell membrane signal transduction

Another key event of radiation acting on tumor cells is that ionizing radiation regulates plasma membrane-related signaling molecules and secondary messengers of neoplasm cells. Based on in vitro assays, Goldkorn [42] and Lammering [43] et al. reported that radiation can activate epidermal growth factor receptor(EGFR) through a non-ligand-dependent pathway and provoke downstream MAPK and PI_3_K pathways, which are powerful mediators of malignant growth and proliferation. In a study on the effects of radiotherapy on gliomas, Park found that radiation switched on the EGFR-mediated p38/Akt and PI_3_K/Akt signaling pathways, which led to increased glioma cell metastasis and invasive ability by up-regulating matrix metalloproteinase 2 (MMP-2) expressions [44]. Alternatively, radiation also promotes cancer cell proliferation, spreading and invasion by upregulating integrin, which is closely related to the diversified biological behaviors of cancer cells [45], or by increasing hypoxia inducible factor (HIF) and activating the hepatocyte growth factor (HGF) /c-Met signal transduction pathway [46]. By contrast, injuries to membrane receptors of tumor cells caused by ionizing radiation can result in a downstream signal transduction pathway imbalance, which causes cellular metabolic pathway confusion and even apoptosis or death. Thus, radiation has dual-functions on cancer cells, not only by damaging DNA to inhibit tumor proliferation but also by changing the expression of molecules associated with invasion and metastasis to promote or restrain tumor development. Accurate descriptions of radiation-sensitive oncology therapeutic signaling pathways will further advance the development of personalized radiotherapy.

### Radiation alters the biological behavior of tumor cells

As normal cells are transformed into cancer cells, they harbor a series of special properties that contribute to the development and progression of the tumor. According to Weinberg’s group, there are ten biological characteristics that are hallmarks of tumors and have led to widespread concern [47]. Based on the relationship between radiation and cancer cells, we integrate the ten properties into six irradiation-related damage scales: 1). The effects of radiation on cell proliferation scale including four of the ten hallmarks (infinite proliferation, escaping growth inhibition, resistance to cell death, and permanent replication); 2). The effects of radiation on invasion and metastasis scale is composed of two of the ten hallmarks (induction of angiogenesis, activation of invasion and metastasis); 3). The effects of radiation on cancer-promoting inflammation scales; the remained three scales involve genomic instability and abnormal energy metabolism what we had discussed above referred to DNA damage and mitochondria, respectively, and immunogenicity which will be discussed later. Therefore, we will kick something around the first three scales. Further elucidating the mechanism of radiation on the iconic biological ability of the tumor will provide a solid theoretical basis for radiotherapy and combined therapy of neoplasm.

#### Effects of radiation on the proliferation scale

Unrestricted proliferation is a major barrier to defeat cancer. The Jumonji domain-containing protein 2B (JMJD2B) is a histone demethylase that promotes the development and progression of gastric cancer, both in mice and human [48]. Kim et al. [49] found that radiation downregulated the level of JMJD2B, which inhibited the expression of cyclin A1 (CCNA1) and, ultimately restrained the proliferation of human gastric cancer AGS cell lines. Additionally, radiation also has a significantly negative influence on squamous cell carcinoma proliferation. As demonstrated by Geraldo et al. [50], high dose rate (HDR) short-range radiation can induce G_2_ / M phase arrest of the radiation-resistant human squamous cell carcinoma A431 cell line by triggering A431 to enter a mitotic death state, which eventually inhibits tumor cell proliferation. This is consistent with the above discussion that radiation-induced DNA damage will result in activation of cell cycle checkpoints and disruption of cell cycle. However, besides depressing proliferation of tumor cells, radiation can also induce ordinary cancer cells to be transformed into induced cancer stem cells (iCSCs), which not only have a strong resistance to radiation but also promote tumor proliferation. According to Lagadec et al. [51], differentiated normal breast cancer cells can be reprogrammed by rays to obtain stemness and develop into induced breast cancer stem cells (iBCSCs), significantly reducing the therapeutic effect. Therefore, to achieve better anti-tumor efficacy, it is necessary to combine radiotherapy with other treatment measures that can inhibit the induction of tumor cells without stemness to tumor stem cells.

#### Effects of radiation on the invasion and metastasis scale

Growing experiments show that radiation can promote tumor epithelial-mesenchymal transition (EMT), which promotes cancer metastasis. EMT is a phenotypic switch that allows tumor cells to detach from intercellular junctions to facilitate metastasis. Jung et al. [52] found that radiation upregulates TGF-β in human lung adenocarcinoma A549 cells, which can induce tumor EMT and enhanced its ability to invade and metastasize. As illustrated by experiments, radiation promotes the invasion and metastasis of colorectal cancer (CRC) cells by enhancing the activity and expression of matrix metalloproteinases (MMPs) [53–55], and studies have elaborated that EMT is related to tumor invasion and metastasis [56–58]. Kawamoto et al. [59] found that radiation promotes a shift of CRCs and also suggested that this radiation-enhanced aggressiveness is associated with morphological and molecular changes, consistent with a shift to a mesenchymal-like phenotype. Confirmed by Zhang et al. [60], when breast cancer MCF-7 cells were exposed to 20Gy rays, expression of the epithelial cell markers CK-18 and E-cadherin was down-regulated, while expression of the stromal cell markers fibronectin and vimentin was up-regulated. The enhancement of invasiveness by radiation in breast cancer MCF 7 cells was confirmed through matrigel invasion experiments, suggesting that radiation promotes breast cancer cell EMT and boosts invasion [60]. In conclusion, the effect of radiation on the phenotype of cancer cells is consistent with EMT conversion, suggesting that combining inhibitors of EMT- activating molecules and radiotherapy may be an effective method to treat cancer.

Induction of angiogenesis is another important force that drives rapid cancer cell metastasis and poor patient prognosis. Tumor angiogenesis is a multifactorial and multi-modal complex process. The changes of vascular endothelial growth factor (VEGF), extracellular matrix-associated protease, adhesion factor and so on contribute to the tumor angiogenesis process. Radiation up-regulates the transcriptional level of VEGF-C in the lung cancer A549 cell line by activating the PI_3_K-Akt-mTOR pathway and increases phosphorylation of 4EBP and eIF4E which as a result promotes tumor neovascularization and endothelial cell proliferation [61]. Consistently, rays promote melanoma angiogenesis by activating TLR4-MYD88-driven inflammatory response [62]. However, radiation inhibits tumor angiogenesis but can easily relapse, which makes joint therapy indispensable. It has been demonstrated by in vitro experiments that radiotherapy plus the autophagy inhibitor 3-methyladenine can more effectively inhibit angiogenesis of the human esophageal squamous cell carcinoma EC9706 cell line compared with radiotherapy alone [63]. In addition, DNA-dependent protein kinase catalytic subunit (DNA-PKcs) inhibitors can eliminate the radiation-induced up-regulation of VEGF and HIF-1α in glioblastoma [64]. Therefore, the effective combination of radiotherapy and DNA-PKcs inhibitors may be a potential union to address glioblastoma under certain circumstances. Illustrating the working mechanism of radiation on the hallmarks of tumor cells will lead to more effective options for cancer therapy.

#### Effects of radiation on the cancer-promoting inflammation scale

Large numbers of immunocytes, cytochemotactic factors, and growth factors that are beneficial to proliferation, invasion, adhesion and angiogenesis promote the initiation and development of cancer in the tumor inflammatory microenvironment (TIM). Tumor resistance to radiation therapy is not only related to the tumor type and tissue distribution but also to the TIM, which has made the relationship between radiation and inflammatory microenvironment a promising topic in recent years [65]. NK cells, immune cells in the TIM, harbor a variety of immunological functions. Early studies manifested that X-ray irradiation enhances the sensitivity of NK cells, which reduces the secretion of exogenous proteins and further retards the growth of malignant tumors [66]. Comparably, T helper cells 17 (Th17) in TIM antagonized Th1 and thus repressed the production of INF-γ [67], however proved by Wang’s team that LDR-induced activation of ataxia telangiectasia mutated (ATM) markedly decreased IL-23 secretion and consequently disturbed Th17 responses [68]. In addition to immunocytes, there are still some regulatory factors in the TIM. At an advanced stage in the tumor development, TGF-β no longer acts as a tumor suppressor, but instead stimulates angiogenesis to some extent, promoting EMT, thereby accelerating tumor development [69]. In summary, precisely distinguishing the composition of the TIM and the diverse responses of the tumors to different doses of radiation are a great help for oncotherapy. It is noteworthy that immune cells or cytokines do not exert a function in the TIM independently but rather in the integrated networks of antineoplastic and oncogenic factors.

### Radiation affects the tumor immune response

Immunotherapy plays an increasingly important role in tumor therapy. Studies of the mechanisms that regulate the immune system and interactions between tumor cells and immune factors have laid a solid theoretical foundation for the treatment of multiple malignant tumors [70]. Moreover, recent studies have shown that tumor radiotherapy is closely interrelated with immune effects, and quantities of experiments have illustrated that radiation affects the division or metastatic biological processes of tumor cells indirectly by impacting the tumor microenvironment and immunogenicity [71, 72]. Parker et al. [73] and Magné et al. [74] found that when a single high dose of radiation was applied to tumor tissue, it regulated the expression of many apoptotic and anti-apoptotic genes by activating the NF-κB signaling pathway and inducing the occurrence and maintenance of immune T cells, B cells and antigen presenting cells (APC). It has been shown that local tumor radiotherapy alters tumor immunogenicity and its interaction with the host, inducing the death of immunogenic antigenic tumor cells and promoting dendritic cells (DC) and T cells to cross-present tumor-derived antigens [75]. At present, the checkpoint inhibitor anti-cytotoxic T-lymphocyte-associated protein 4 (anti-CTLA-4) and anti-programmed death-1 (anti-PD-1) are representative of the tumor immunotherapy program and have been widely studied. In 2011 and 2013, they were applied in clinical treatment respectively and their combinations with radiotherapy bring hope to tumor patients. CTLA4 blockade predominantly restrains T regulatory cells (Tregs) to increase the proportion of CD8^+^T cells (CD8/Treg). Anti-PD-1/PD-L1 relieves T cell exhaustion to mitigate depression in the CD8/Treg ratio and expand the distribution of oligo-clonal T cells. Irradiation facilitates the agonist diversity of the Toll-like receptors (TLRs) and maintenance or amplification of cancer cell immunogenicity.

#### Ray-enhanced anti-CTLA-4 immunotherapy

CTLA-4 is a leukocyte differentiation antigen located on the T cell membrane and functions as a transmembrane receptor. When it binds to a co-stimulatory molecule (B7) that is situated at the surface of APC, it induces T cells to be nonresponsive to cancer cells. CTLA-4 can down-regulate or terminate T cell- mediated immune responses by inhibiting T cell activity, which is consistent with the notion that CTLA-4 is a negative regulator of the anti-tumor immune response, demonstrating that antibody blockade of CTLA-4 could result in antitumor immunity in preclinical models. Although CTLA-4 antibodies bear good prospects, they are still limited to intrinsically immunogenic tumors. However, radiation therapy combined with anti-CTLA-4 monoclonal antibody treatment has made great progress in anti-tumor practice. Using a mouse model of breast cancer, Demaria et al. [76] found that the combination of local RT and CTLA-4 blockade significantly inhibited the growth and metastasis of primary tumors and produced better response outcomes in patients with spontaneous metastasis and low-immunogenicity breast cancer compared to treatment alone, and the intrinsic mechanism may be that combination therapy induces more CD8^+^T cells with enhanced activity, which is propitious to CTLA-4 antibody-mediated tumor immunotherapy treatment. Additionally, a similar conclusion was drawn in colon cancer therapy by Son et al. [77]. Obviously, stimulating the immune defense system of the organism and improving the body’s immune function can improve the efficiency of treating cancer.

#### Achievements of radiation combined with anti –PD-1/PD-L1 immunotherapy

Similar to anti-CTLA-4 immunotherapy, anti-PD-1/PD-L1 monoclonal antibodies also play a positive role in tumor therapy and achieve greater progress when combined with radiotherapy. Antibodies targeting the PD-1/PD-L1 checkpoint reverse immunocyte inhibition by blocking recognition of PD-1 located on Tregs or NK cells and PD-L1 expressed on tumor cells, and then restore the immunologic surveillance and anti-tumor abilities. PD-1 is a well-defined immune suppressor. Nvolumab and Pembrolizumab are PD-1 monoclonal antibodies (mAbs) that have been approved for clinical use with a lot achievement in improving efficacy [78, 79]. Zeng et al. [80] found that compared to a single treatment-alone, combination therapy with radiation and the PD-1 antibody not only increased the survival of mice implanted with intracranial gliomas by increasing the ratio of Tregs but also induced immune memory responses. Analogously, according to Sharabi et al. [81], compared with the control group, radiotherapy combined with PD-1 immunotherapy increased the infiltration of intratumoral T cells in established B16 and 4 T1 tumors. Furthermore, programmed death-ligand 1 (PD-L1) is also a potential target for immunotherapy, and atezolizumab, durvalumab and avelumab are PD-L1 antibodies that are currently the furthest in clinical development [82–84]. As certified by Deng et al. [85], ionizing radiation plus anti-PD-L1 antibody can activate cytotoxic T cells, which can reduce tumor-infiltrating bone marrow-derived inhibitory cells (MDSCs) through the cytotoxic actions of TNF, ultimately releasing T cell inhibition. Based on the above evidence, radiation can significantly enhance the effects of immunotherapy. Radiation combined with PD-1/PD-L1 monoclonal antibodies may have great promise and therapeutic potential for a variety of malignancies.

Although these monoclonal antibodies are currently the most promising immunotherapeutic anti-tumor drugs, their high cost of production and side effects, such as pneumonia, diarrhea and skin diseases, have limited their development which leaves way for PD-1/PD-L1-targeting small molecule inhibitors to a broader field [86–88]. The pharmacokinetic behavior of small molecule inhibitors is highly controlled and well complementing the clinical shortcomings of macromolecular mAbs. CA-170, a class of small molecule inhibitors of PD-1/PD-L1 that can be orally administered, is already approved by the Food and Drug Administration (FDA) in the United States and is undergoing phase I clinical validation [89]. However, the number of PD-1/PD-L1 micro molecule inhibitors reported to date is very low due to the finite information on the interaction between PD-1 and PD-L1, and the impact of radiation on its therapeutic effect still remains elusive.

#### TLR-mediated immunologic effects of RT

TLRs are a class of important protein molecules that are involved in innate immunity, which can recognize damage-associated molecular patterns (DAMP), such as “danger” signal molecules released in the milieu upon RT. Tumor radiation results in upregulation or generation of tumor-associated antigens (TAA) and DAMPs including HMGB1 and HSPs [90, 91], which served as agonists of TLRs and thus primed “immunogenic cell death” via TLR-mediated signaling pathways. Under irradiation, systemic treatment of R848, a TLR7 agonist, leads to a dramatic boost of the anti-tumor efficiency in T cell lymphoma mouse models [92]. Alternatively, both Milas [93] and Mason [94] demonstrated that CpG enhances the immunologic effects of RT by activating TLR9 in fibrosarcoma mouse tumor models. Clinically, the combination of RT together with TLR modulators has been a major breakthrough for patients bearing esophageal squamous cell carcinoma or breast cancer [95, 96]. Irradiation, therefore, is a potential treatment for performing in-situ immunization because it can initiate the release of TAAs and stimulate APCs via endogenous release of DAMPs or TCR agonists.

Moreover, radiation maintains tumor specific molecular immunity and enhances the secretion of tumor cytokines (CXCL9, CXCL10 and CXCL16) to promote tumor immune recognition and T cell infiltration and inducing immunogenic cell death (ICD) [97]. RT is also able to promote chemokine-mediated effector T cells recruitment around tumor cells and trigger a distant effect (or bystander effect), which passes the radiation signal factor through the circulatory system to distant tumor cells or tissues [5, 98]. In summary, radiation influences the progression of cancer extensively, and its anti-tumor effects are dominant.

### Radiation-related tumor-promoting response

Although radiation largely produces toxic effects on tumors from many aspects, we cannot deny the side effects of radiation, which can promote the occurrence and development of cancer. Experiments have shown that radiation induced the expression of TGF-β in liver non-parenchymal cells (NPCs), which is able to promote the invasion and metastasis abilities of hepatocellular carcinoma cells (HCC) [99]. High doses of radiation are known to prime increased TGF-β1 secretion by irradiated stroma, which is considered to be a larvaceous stimulatory agent in the late phases of carcinogenesis and metastasis dissemination [100]. Parker et al. [73] unearthed that the non-specific damage caused by radiation to normal cells increased the patient’s lymphocyte damage and functional inhibition, which allowed immunogenicity-compromised tumor cells to escape immune surveillance. Worse still, radiation also activates immunosuppressive factors stimulating inhibitory immune cells such as Tregs and M2 macrophages that inhibit T and NK cell-mediated immune responses, thus, enhancing tumor aggressiveness [101–103]. Besides, changes in the tumor microenvironment in turn affect radiation-induced tumor repression. Hypoxia-triggered accumulation of adenosine, an essential tumor-microenvironmental factor (TMF), not only promotes tumor proliferation and angiogenesis directly but thwarts the RT-elicited antitumor immune responses [97]. Summarized by Vaupel et al. [104], apart from ADO, other TMFs, such as lactate accumulation, extracellular acidosis, VEGF overexpression and phosphatidylserine externalization driven by hypoxia, may also sabotage innate or therapeutically triggered antitumor immune responses. Therefore, the adoption of precise radiotherapy regimens in combination with immune checkpoint blockers [81, 105] and antibodies or molecule inhibitors targeting TMF is a promising attempt that will regulate systemic immunity response and ultimately achieve optimized therapeutic effect.

## Conclusion

This review focuses on the relationship between radiation and tumor biology to clarify how radiation affects the biological structure and behavior of neoplasm cells, which is helpful for clinicians to achieve effective radiotherapy for maximal eradication of cancer cells while minimally killing normal ones. Although radiotherapy is a double-edged sword, with the development of molecular biology and radiation oncology, radiotherapy plays an increasingly important role in the treatment of cancer and lays a solid foundation for its clinical application. Radiation can damage DNA, organelles, and cell membrane or change the immunogenicity and microenvironment of tumor cells to regulate tumor cell apoptosis, proliferation, differentiation, migration and biological functions; additionally, it can activate a variety of signaling pathways to inhibit the body’s immune response and promote tumor development. Thus, the dual effect of radiation and immunotherapy on the tumor will be a major topic in the future treatment of cancer. To obtain high-precision as well as low-damage radiotherapy efficacy, combining precise radiotherapy and chemotherapy or immunotherapy and other treatment methods is particularly attractive.

## Abbreviations

Anti-CTLA-4: Anti- cytotoxic T-lymphocyte-associated protein 4
Anti-PD-1: Anti-programmed death-1
APC: Antigen presenting cells
ATM: Ataxia telangiectasia mutated
CCNA1: Cyclin A1
CRC: Colorectal cancer
Cyt-c: Cytochrome C
DAMP: Damage-associated molecular patterns
DC: Dendritic cells
DNA-PKcs: DNA-dependent protein kinase catalytic subunit
DSB: Double-strand breaks
EGFR: Epidermal growth factor receptor
EMT: Epithelial-mesenchymal transition
ERS: Endoplasmic reticulum stress
FDA: Food and Drug Administration
HCC: Hepatocellular carcinoma cells
HDR: High dose rate
HGF: Hepatocyte growth factor
HIF: Hypoxia inducible factor
iBCSCs: Induced breast cancer stem cells
ICD: Immunogenic cell death
iCSCs: Induced cancer stem cells
IFN-1: Interferon-1
JMJD2B: Jumonji domain-containing protein 2B
mAbs: Monoclonal antibodies
MDSCs: Marrow-derived inhibitory cells
MMPs: Matrix metalloproteinases
mtDNA: Mitochondrial DNA
NHEJ: Non-homologous end joining
NPCs: Non-parenchymal cells
Ptpn14: Protein tyrosine phosphatase non-receptor Type 14
RNS: Reactive nitrogen species
ROS: Reactive oxygen species
SREBP: Sterol regulatory element binding protein
SSB: Single-strand broken
TAA: Tumor-associated antigens
Tregs: T regulatory cells
UPRs: Unfolded protein response
VEGF: Vascular endothelial growth factor
VEGF-C: Vascular endothelial growth factor-C
